# A lightweight neural network with multiscale feature enhancement for liver CT segmentation

**DOI:** 10.1038/s41598-022-16828-6

**Published:** 2022-08-19

**Authors:** Mohammed Yusuf Ansari, Yin Yang, Shidin Balakrishnan, Julien Abinahed, Abdulla Al-Ansari, Mohamed Warfa, Omran Almokdad, Ali Barah, Ahmed Omer, Ajay Vikram Singh, Pramod Kumar Meher, Jolly Bhadra, Osama Halabi, Mohammad Farid Azampour, Nassir Navab, Thomas Wendler, Sarada Prasad Dakua

**Affiliations:** 1 Hamad Medical Corporation, Doha, Qatar; 2 Hamad Bin Khalifa University, Doha, Qatar; 3 Qatar University, Doha, Qatar; 4 Technische Universität München, Munich, Germany; 5 C. V. Raman Global University, Bhubaneswar, India; 6 German Federal Institute for Risk Assessment (BfR), Berlin, Germany; 7 Wake Forest Baptist Medical Center, Winston-Salem, USA

**Keywords:** Computational biology and bioinformatics, Image processing

## Abstract

Segmentation of abdominal Computed Tomography (CT) scan is essential for analyzing, diagnosing, and treating visceral organ diseases (e.g., hepatocellular carcinoma). This paper proposes a novel neural network (Res-PAC-UNet) that employs a fixed-width residual UNet backbone and Pyramid Atrous Convolutions, providing a low disk utilization method for precise liver CT segmentation. The proposed network is trained on medical segmentation decathlon dataset using a modified surface loss function. Additionally, we evaluate its quantitative and qualitative performance; the Res16-PAC-UNet achieves a Dice coefficient of 0.950 ± 0.019 with less than half a million parameters. Alternatively, the Res32-PAC-UNet obtains a Dice coefficient of 0.958 ± 0.015 with an acceptable parameter count of approximately 1.2 million.

## Introduction

Computed Tomography (CT) scan is a non-invasive medical imaging technique for obtaining 3D high-resolution images of different organs^1^. Interventional radiologists rely heavily on CT scans for the diagnosis and treatment of cancer and metastasis in the visceral organs (e.g., hepatocellular carcinoma (HCC)). Moreover, CT imaging is an alternative to MRI for imaging patients with metallic implants and pacemakers. Specifically, CT scans are necessary for localizing tumors, examining tumor shapes, and estimating tumor volume in different liver segments, thus playing a vital role in the diagnosis and treatment planning of HCC.

Analysis of abdominal scans is for detecting liver cancer and other visceral diseases. Conventionally, the radiologists manually delineate the region of interest (ROI) (i.e., liver and tumors) to measure the cancer spread and plan appropriate treatment. Outlining the liver and its tumors allows the surgeons to plan treatment that minimizes the damage to healthy liver tissues. The delineated CT scan can further be registered with other imaging modalities (e.g., Ultrasound), producing enhanced visualization for image-guided surgeries. However, the manual delineation of medical images is tedious, and operator-dependent^2^. Automatic segmentation algorithms with low disk and memory utilization can serve as an effective alternative to the manual delineation by generating the segmentation masks of the liver and its tumors based on the input CT scans. These automatic segmentation algorithms can save both time and effort of interventional radiologists and surgeons, allowing them to focus more on treatment planning and surgeries.

The automatic segmentation algorithms face several challenges due to the nature of CT scans (e.g., electronic noise, varying axial resolution, etc.). In addition to these challenges, the segmentation algorithms may need to work in a disk and memory-constrained environment in hospitals to maximize their applicability and usability. To elaborate further, several scenarios in a clinical space require lighter segmentation models: (1) Efficient intra-procedural image fusion to improve visualization (across imaging modalities) requires automated liver tumor segmentation techniques for planning appropriate treatment procedures. Lightweight CT segmentation tools enhance the chances of being deployed on clinical machines (in operation theaters) to help reduce total procedure time, where the patient may be under sedation/anesthesia or being exposed to chemoradiation. (2) Turnaround time (TAT) is an essential quality indicator of radiology services, especially in the emergency setting. Segmentation models that maximize segmentation accuracy could be crucial in improving TAT and clinical workflows if they have lower computational requirements (like disk utilization).

Over the years, many conventional^3–5^ and deep learning-based^6–8^ segmentation algorithms have been proposed to overcome the challenges in CT scans and maximize segmentation accuracy. However, the methods have not emphasized maximizing performance in disk and memory-constrained environments. The conventional segmentation techniques are based on region growing^4,5,9^, thresholding^10,11^, watershed^12^, active contours^13,14^, clustering^11,15^, graph cut^16,17^, etc. These techniques may have application-specific advantages, but their dependence on primitive image features (e.g., pixel intensities and edge maps) significantly impacts their robustness and generalization capability. Recently, deep learning-based techniques have gained significant attention for liver CT segmentation because of their improved accuracy, automation, and robustness^8^.

The neural network-based segmentation overcomes the limitations of the conventional segmentation methods by automatically extracting relevant features using convolutional kernels in a data-driven manner. UNet^7^ is one of the popular architectures for biomedical image segmentation. One of the main advantages of the UNet over the preceding fully convolutional networks (FCNs) is that it has a dedicated decoder for stage-wise construction of the segmentation masks. Additionally, the UNet has skip connections to improve information/gradient flow and alleviate the loss of spatial information caused by repeated pooling operations. Initially, UNet was proposed to segment 2D biomedical images, Çiçek et al.^18^ extend the UNet architecture for volumetric segmentation of medical images by replacing 2D kernels with 3D kernels in convolution layers. Milletari et al.^19^ present the VNet architecture that uses convolutions with strides (i.e., alternative to max-pooling) for down sampling image resolution and de-convolution (i.e., alternative to bi-linear upsampling) to upscale encoded features for generating the segmentation masks. Like UNet, the VNet employs skip connections to improve gradient flow through the network. However, the UNet and VNet architectures may not be suitable for segmenting noisy CT scans with poor contrast because they lack network logic that can capture ambiguous anatomical boundaries (i.e., liver overlapping with other organs) and small ROIs (e.g., liver tumors).

In recent years, custom encoder-decoder architectures with custom modules have been proposed to overcome the shortcomings of vanilla 3D UNet. Han et al.^20^ present a deep convolutional neural network that uses short residual skip connections within the backbone and long skip connections between the encoder and decoder to improve feature propagation. The model achieves acceptable segmentation accuracy but has a large parameter count and longer inference times. Sun et al.^21^ introduce a multi-channel fully convolutional network (MC-FCN) for segmenting liver tumors from multi-phase contrast-enhanced CT scans by utilizing phase information to generate high-level fused features. The critical limitation of MC-FCN is the lack of multi-phase CT data, limiting the method’s applicability in clinical settings. Zhang et al.^22^ propose a prior propagation module (PPM) to learn the spatial priors of the pancreas on different axes. Then, a scale-transferable feature fusion module (STFFM) is employed to generate rich feature fusion. Resulting DCNN outperforms other architectures for pancreatic segmentation but contains nearly 25 million parameters (i.e., high disk usage). Zhang et al.^23,24^ have also introduced a network that captures and combines information from different modality MRI scans to segment three different regions of brain tumors effectively. The work is limited by minimal discussion on parameter count, disk utilization, inference time, and evaluation with brain CT data.

Variants of UNet^25,26^ have also been proposed for segmenting liver in CT scans. Li et al.^8^ introduce H-DenseUNet, which uses 2D Dense-UNet for extracting intra-slice information and 3D Dense-UNet for aggregating volumetric details. Seo et al.^27^ present a modified UNet (m-UNet) architecture with a residual de-convolution module over the skip connections. The resultant network effectively combines the 3D voxel information of the encoder and passes it to the decoder to achieve high liver segmentation accuracy. One significant limitation of H-DenseUNet and m-UNet is the presence of numerous parameters, which results in prolonged training, higher memory footprint, and model size, thus impacting their usability on clinical computers. Jha et al.^28^ propose the Res-UNet++ architecture using squeeze and excitation principle in the encoder and attention mask in the decoder to propagate relevant features for the liver segmentation task. Furthermore, the network employs an atrous spatial pyramid pooling module at the bottleneck to generate multi-scale high-level features. Jha et al.^28^ have highlighted in their discussion that the increased performance of the Res-UNet++ is at the cost of increased network parameters. Ibtehaz et al.^29^ propose the Multi-Res-UNet that combines features extracted with kernels of different sizes using residual connections in the network backbone. Further, the skip-connections contain a sequence of convolutional layers to decrease the semantic gap between the encoder and decoder. Lou et al.^30^ suggest another network, DC-UNet, by modifying the multi-res block, introduced by Ibtehaz et al.^29^ with a dual-channel block to enhance the capability of residual connections for extracting multi-scale features. One common limitation of Res-UNet++, Multi-Res-UNet, and DC-UNet is the use of 2D convolutions in their implementation, which does not utilize the axial information of CT scans. Modifying their implementation with 3D convolutions exponentially increases the network parameters, preventing their training due to GPU memory constraints. Throughout our review, we have observed that UNet and its variants double the feature map width at every encoder stage, resulting in large parameter counts, making them infeasible for deployment on machines with disk and memory constraints.

We have also identified two fundamental limitations of the UNet. Firstly, UNet employs skip connections between the encoder and the decoder resulting in the duplication of low-resolution feature maps. The low-level extracted features in the early layers of the encoder propagate through the network backbone. Simultaneously, the skip-connections transfer the same feature from the encoder to the decoder, resulting in feature redundancy and causing smoothing of anatomical boundaries. Secondly, the high-level features learned in the deeper layers of the encoder lack the organ boundary information due to repeated pooling operations, thus impacting the segmentation accuracy^27^.

In this paper, we propose a novel neural network architecture inspired by Thin-UNet^31^, namely, the Res-PAC-UNet, that overcomes the limitation of UNet and its variants for liver CT segmentation. Specifically, our method aims to achieve high liver segmentation accuracy while utilizing less network parameter and disk space. Fundamentally, we make the following contributions:

1. We propose tuned backbones with residual connections and fixed-width that minimize the parameter count of the network, while improving gradient flow and segmentation performance (Methodology: Network Architecture).
2. We introduce a Pyramid Atrous Convolution (PAC) module over the skip connections of the encoder to extract multiscale volumetric features, assisting the network in constructing liver masks from CT scans with poor anatomical boundaries and contrast (Methodology: Pyramid Atrous Convolution Module).
3. We modify the surface loss function proposed by Kervadec et al.^32^ by incorporating the combo loss, allowing the loss function to quantify the discrepancies between the network prediction and ground truth (Methodology: Loss Functions).
4. We empirically evaluate the impact of the loss functions and PAC module on the segmentation accuracy of the Res-PAC-UNet and other architectures in the UNet family. Furthermore, we compare the segmentation performance of the Res-PAC-UNet with the state-of-the-art for liver CT segmentation models^28–30^ (Results and Discussion).

To the best of our knowledge, this work presents significant advantages over the existing work in the literature by emphasizing lower parameter count, smaller model size, and usability of the model without compromising the segmentation accuracy.

The remainder of the paper is structured as follows: “Introduction” Section describes our proposed methodology, including the neural network architectures, PAC module, and the loss function. “Experimental setup” Section explains the experimental setup by providing information about the dataset, preprocessing, evaluation metrics, and implementation. “Results and discussion” Section highlights the results of the empirical study and discusses the critical observations and findings. Finally, “Conclusion” Section summarizes our contribution and concludes the paper.

## Proposed methodology

### Network architecture

**Figure 1:**
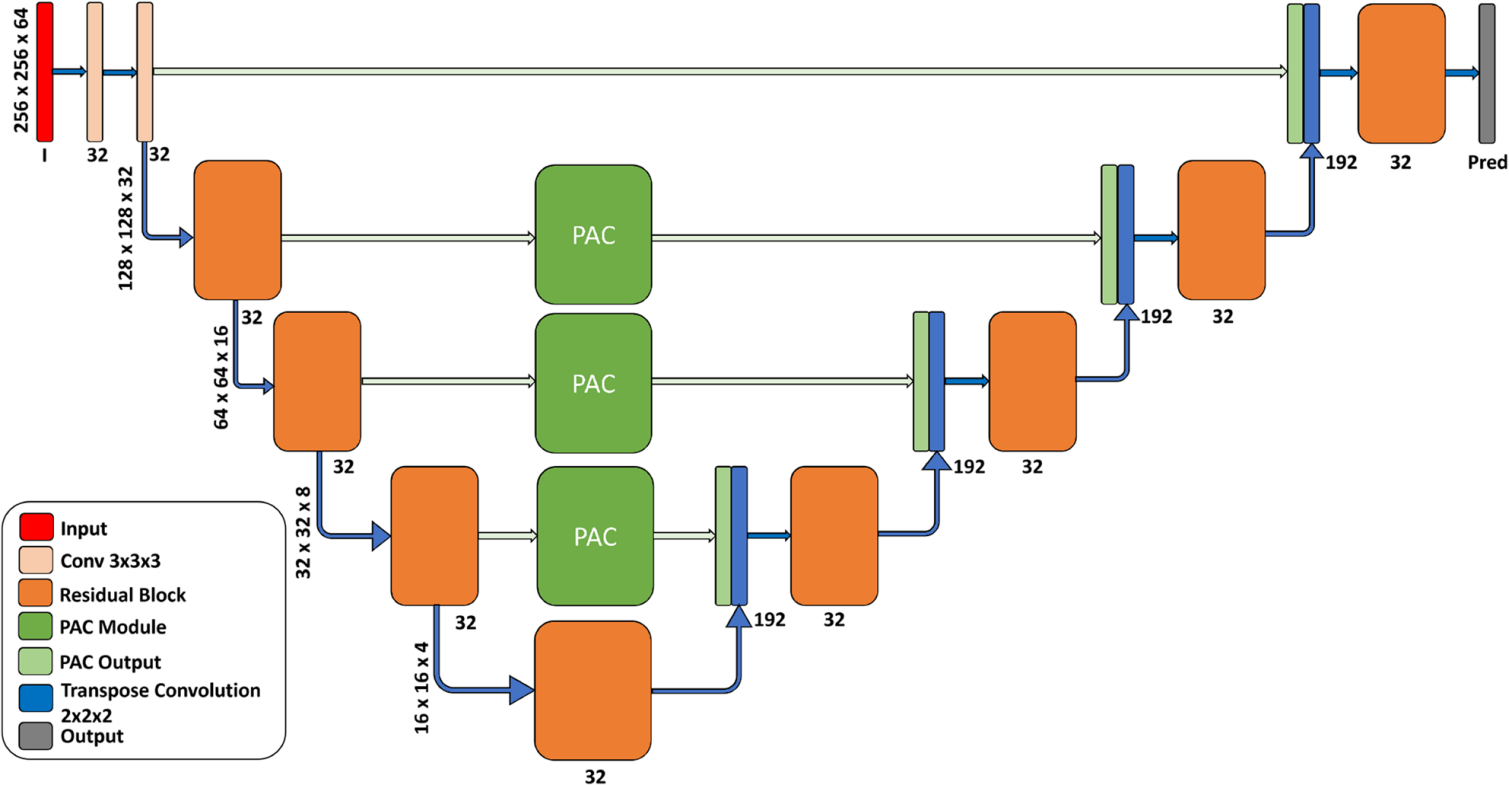
Lightweight Res32-PAC-UNet architecture for high accuracy liver CT segmentation.

Our Res-PAC-UNet architecture (Fig. 1) overcomes the major limitations of the UNet and its variants for liver CT segmentation. Firstly, Res-PAC-UNet has a tuned backbone with constant feature width (K) and residual blocks to minimize the parameter count and the memory footprint of the network while improving the information and gradient flow. The constant feature width (K = 16 or = 32) prevents the exponential increase in features (from 32 to 256) in the deeper layers of UNet. We have selected K (i.e., 32) based on the initial feature width of the UNet^7^. We have also trimmed the K to half of the initial width of the UNet (i.e., 16) to understand its impact on segmentation accuracy. Unfortunately, We could not train the Res-PAC-UNet with higher values of K due to limited GPU memory. Secondly, we overcome the problem of redundant features and loss of edge information in deeper feature maps by proposing an intuitive solution of generating features of different scales before transferring them to the decoder. To compute multi-scale volumetric features at different encoder levels, we place PAC modules (“Pyramid atrous convolution module” Section) over the skip connections. We avoid placing the PAC modules at the top skip connection to minimize GPU memory required by the high resolution of the feature maps.

**Figure 2:**
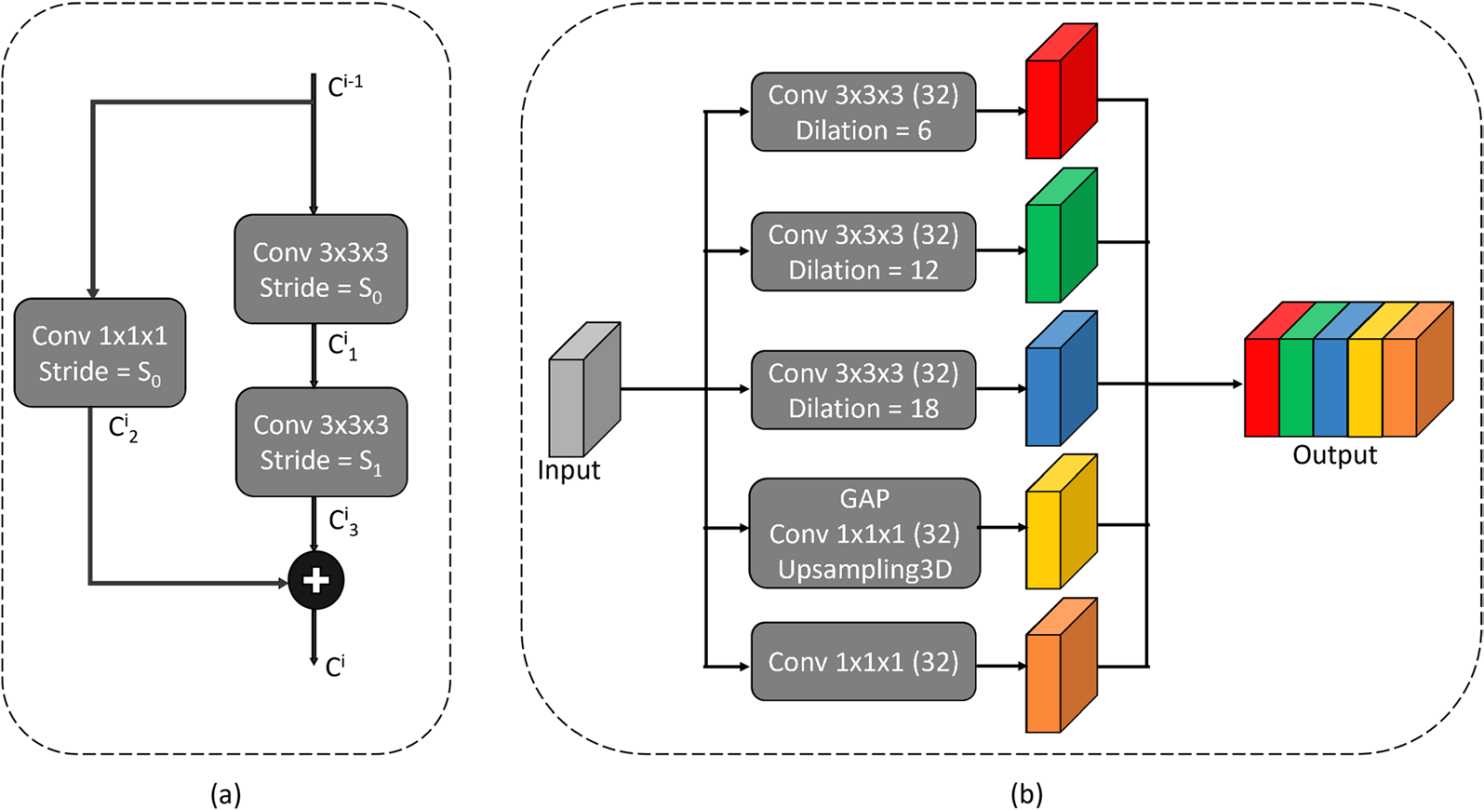
(**a**) Residual block employed in the backbone for improving information and gradient flow. (**b**) PAC module for capturing multi-scale volumetric features at different levels of the encoder.

Additionally, The residual blocks replace the convolutional blocks and perform downscaling of the input feature maps by employing strided convolutions. Figure 2 shows the residual block utilized in the tuned backbone. Initial convolutions in the encoder residual blocks operate a stride of 2 ($s_0=2$) to downscale the feature map resolution by half. On the other hand, the decoder employs transpose convolutions (i.e., deconvolution) to upscale the feature map resolution and regular convolutions with a stride of 1 in the residual blocks. The regular convolutional operation and residual blocks in the proposed backbone can be mathematically expressed as follows:

1 $$\begin{aligned} Conv_{\_m\times m\times m}(x,s,K;\theta ) = f(w^j \circledast _s x + b^j), \forall 1\le j\le K, w^j \in \theta , b^j \in \theta , \end{aligned}$$

where *x* is the input feature map, *s* is the stride of the convolution, *K* is the number of kernels, *m* is the dimension of the kernels; $\theta$ contains the weights and biases of all kernels, *f*(.) is the activation function applied to the result of the convolution, $\circledast _s$ is the strided convolution operation, $w^j$ and $b^j$ are the weight and bias of the *j*th kernel, respectively. Based on this definition of the convolution operation, the residual block can be expressed as:

2 $$\begin{aligned} \begin{aligned}{}&c^{i}_1 = Conv_{\_m\times m\times m}(c^{i-1},s_0,K;\theta ^{i}_1),\\&c^{i}_2 = Conv_{\_m\times m\times m}(c^{i-1},s_0,K;\theta ^{i}_2),\\&c^{i}_3 = Conv_{\_m\times m\times m}(c^{i}_1,s_1,K;\theta ^{i}_3),\\&c^{i} = c^{i}_2 \oplus c^{i}_3, \end{aligned} \end{aligned}$$

where $c^{i-1}$ and $c^{i}$ are the input and output of the residual block, respectively. $c^{i}_1$, $c^{i}_2$, $c^{i}_3$ are the outputs of the three convolution operations. $\oplus$ is the element-wise addition operation.

#### Empirical comparison of neural networks

We evaluate our proposed models’ segmentation performance and disk utilization by conducting an extensive experimental study with the original UNet as the baseline model. Then, we tune the UNet backbone by shrinking the feature width of the first layer, thereby every successive layer of its encoder, to decrease the overall parameter count. We name the resulting model Tuned-UNet. Subsequently, we add the PAC module to the proposed tuned Res-UNet (Res-PAC-UNet) to measure its performance impact. Finally, we modify the Thin UNet architecture by adding PAC modules to compare its performance with the Res-PAC-UNet architecture. For Res-PAC-UNet and Thin-PAC-UNet, we are limited to constant feature widths of 16 and 32 in the backbones due to memory constraints on the GPU. Furthermore, we include Res-UNet++^28^, Multi-Res-UNet^29^, DC-UNet^30^, and TMD-UNet^33^ architectures proposed for liver CT segmentation in our study to establish a thorough comparison with the literature.

### Pyramid atrous convolution module

Zhao et al.^34^ propose a Pyramid Scene Parsing (PSP) module to improve semantic segmentation performance by enhancing contextual relationships between the image regions and serving as a global contextual prior. The PSP module extracts multi-scale contextual features by performing max pooling operations at different scales, followed by 1$\times$1 convolutions and concatenation. However, the PSP results in loss of spatial information due to pooling operations. Chen et al.^35^ overcome the limitation of the PSP module in the DeepLabV3 architecture by replacing the max pooling operation with atrous convolution. The resulting Atrous Spatial Pyramid Pooling (ASPP) utilizes convolution with different dilation rates to capture varying fields of view in the feature maps, thereby generating multi-scale volumetric features and controlling the receptive field of the network. It can be observed that the 3$\times$3 convolution in the ASPP module degrades to a 1$\times$1 convolution for edge pixels of a feature map due to the large dilation between the convolutional filter’s weights. Chen et al.^35^ recover the missing edge pixel information by using global average pooling (GAP) followed by upsampling.

This paper proposes the PAC module based on ASPP to segment abdominal 3D CT scans. Fundamentally, PAC is a 3D extension of ASPP with lower dilation rate convolutions (i.e., 6, 12, and 18). We drop high dilation rate convolutions (e.g., *dilation rate = 24*) because the overall size of the kernel becomes similar to the dimension of feature maps in the deeper layers of the encoder, helping to decrease the parameter count of the PAC module. These convolutions may not be helpful because they may capture features from different corners of the CT scan rather than emphasizing the liver region. The lower dilation 3D convolutions allow PAC to extract organ-specific multi-scale volumetric features. These features allow the network to utilize essential intra- and inter-slice information to differentiate the liver from the background. The PAC module is placed at the deeper skip connections of the tuned Res-UNet backbone to pass multi-scale features from multiple levels of the encoder, thus preventing duplication of low-level features and smoothing of anatomical boundaries. Finally, the decoder utilizes multi-scale information when upsampling the feature maps at different stages to construct liver segmentation masks effectively. Figure 2 shows the components of the PAC module. The dilated convolutions used in the PAC module can be mathematically expressed as:

3 $$\begin{aligned} \begin{aligned} Conv_{d_{\_m\times m\times m}}(x,s,r,K;\theta ) = f(w^j_r \circledast _s x + b^j_r), \forall 1\le j\le K, w^j_r \in \theta , b^j_r \in \theta , \end{aligned} \end{aligned}$$

where *r* is the dilation rate, $w^j_r$ and $b^j_r$ are the weight and bias of the dilated *j*th kernel, respectively. The rest of the parameters have the same meaning as in the regular convolution defined previously. Based on this definition of the dilated convolution operation, the PAC module can be given by:

4 $$\begin{aligned} \begin{aligned}{}&pac_1 = Conv_{d_{\_3\times 3\times 3}}(I,s_0,6,32;\theta _1),\\&pac_2 = Conv_{d_{\_3\times 3\times 3}}(I,s_0,12,32;\theta _2),\\&pac_3 = Conv_{d_{\_3\times 3\times 3}}(I,s_0,18,32;\theta _3),\\&pac_4 = Conv_{1\times 1\times 1}(I,s_0,32;\theta _4),\\&pac_5 = Upsample3D(Conv_{1\times 1\times 1}(GAP(I),s_0,32;\theta _5)),\\&O = pac_1 \frown pac_2 \frown pac_3 \frown pac_4 \frown pac_5, \end{aligned} \end{aligned}$$

where *I* and *O* are the input and output of the PAC module, respectively. $pac_i,1\le i\le 5$ are the outputs of the five sub-operations within PAC. $\frown$ is the tensor concatenation operation. *GAP* represents the global average pooling operation. *Upsample*3*D* rescales the feature map to the same dimension as the $pac_i,1\le i\le 4$.

### Loss function

A loss function is an essential component of a neural network training procedure because it effectively quantifies the discrepancies between the ground truth and prediction. For the image segmentation task, the neural network needs to learn the ROIs’ area, statistical distribution, and boundaries.

#### Modified surface loss

Kervadec et al.^32^ propose a boundary loss function using the distance metrics of the shape contours for quantifying the anatomical boundary errors. The boundary loss is described using a graph-based optimization for estimating the gradient flow for curve evolution. Different components of boundary loss are the regional softmax probabilities of the pixels ($\Omega$) in the predicted segmentation mask ($M_\theta$) and the level-set function pre-computed on the ground truth ($\phi _G$).

5 $$\begin{aligned} BL(\Omega ) = \int _{\Omega }^{} \phi _G(p)M_\theta (p)dp. \end{aligned}$$

here the boundary loss is computed by multiplying probabilities in the prediction with the level-set function of the ground truth and integrating the result over all the pixels. Kervadec et al.^32^ highlight that the combination of boundary loss with region-based loss function (surface loss) provides up to 8% performance improvement in Dice coefficient. We modify the surface loss function by replacing the generalized Dice loss with combo loss (sum of Dice loss and focal loss) that emphasizes the ROIs’ class and area distribution, aiming to improve the class accuracy metrics. Furthermore, we propose an alternative weight shifting strategy, shifting the weight from 0.99 to 0.25 on the combo loss and 0.01 to 0.75 on the boundary loss. Initial increased weight on combo loss ensures that the network learns the area and statistical distribution of the liver in the earlier epochs. As the weight shifts towards the boundary loss in later epochs, the network is trained to learn the anatomical boundaries of the liver. To add stability to network training, the suggested weight shifting strategy ensures that the combo loss has a fair portion of the net weight at the end of the training.

## Experimental setup

### Dataset and pre-processing

We employ the liver CT scans provided in the medical segmentation decathlon^36^ to train our models. The liver segmentation challenge contains 201 contrast-enhanced CT scans divided into the training (131 scans) and test (70 CT scans) sets. The spatial dimension of the CT scans is 512$\times$512, with the number of slices in the range of (50, 1100). The CT scans belong to the patients suffering from HCC and other liver diseases resulting from lung, breast, or colorectal cancers. The liver decathlon dataset was acquired at the IRCAD Hôpitaux Universitaires, Strasbourg, France, and shares a small number of CT scans with the 2017 Liver Tumor Segmentation (LiTS) challenge^37^. The ground truths of the CT scans in the test set are undisclosed because of ongoing community challenges. For this reason, we split the original training set of the dataset and performed training with 101 scans and testing with 30 scans. Despite the quality measures taken during the acquisition, the CT scans have poor contrast, overlapping anatomical boundaries, noise, and significant variations in axial resolution, making the image pre-processing, neural network training, and evaluation of liver CT scans challenging.

**Figure 3:**
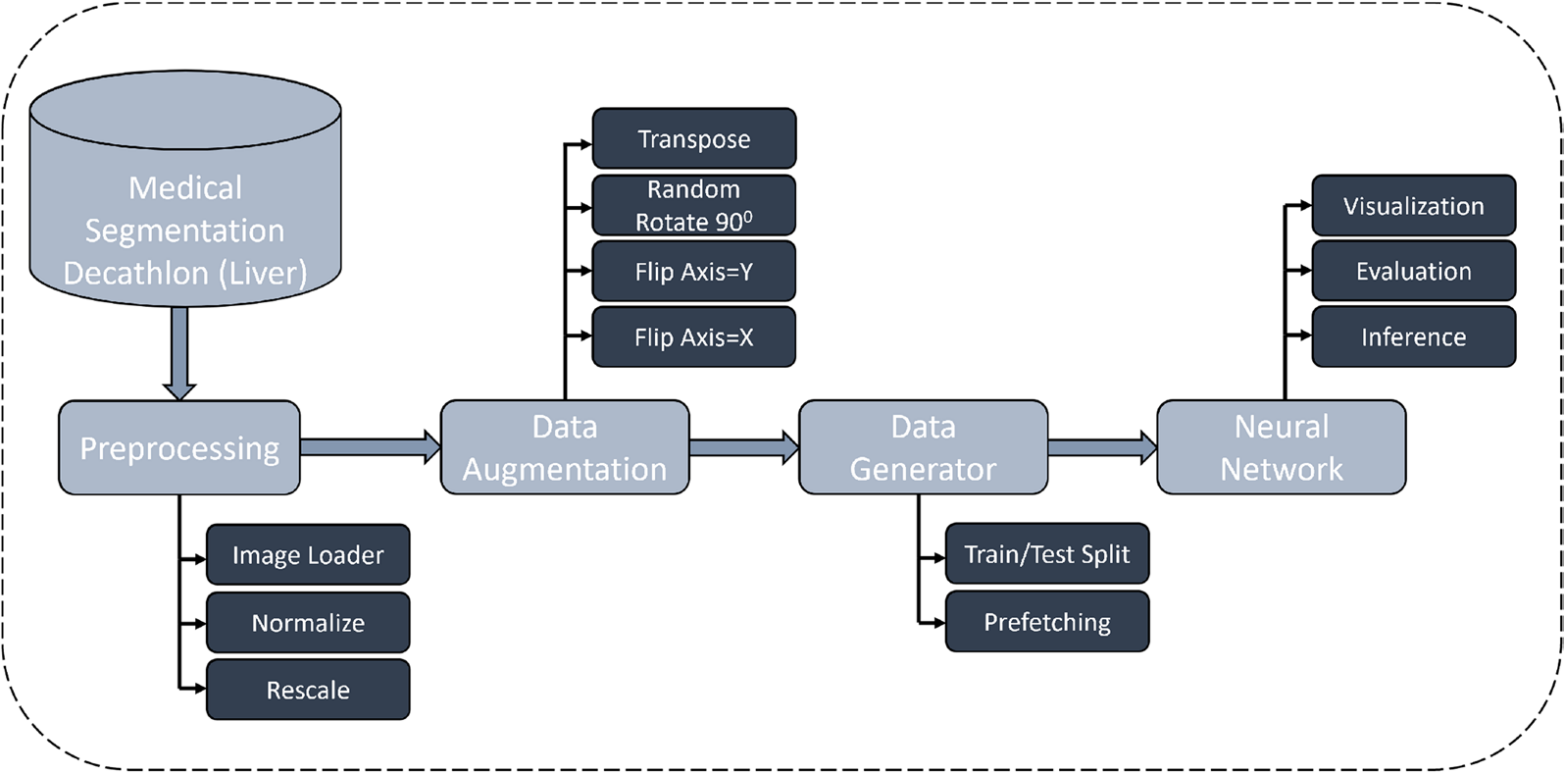
Proposed deep learning framework for training and inference of lightweight liver CT segmentation models.

Figure 3 shows the deep learning CT segmentation framework. In the pre-processing stage, we read the file using the Nifti loader and cap the image intensities of all the scans in the range [− 500, 500] HU. Next, we perform min-max normalization to recompute the image intensities to [− 1, 1]. One significant challenge while designing networks for 3D CT is VRAM consumption. We resize the spatial dimensions of the input scans to 256$\times$256 and resample 64 slices from the liver region of each scan to minimize the VRAM footprint of the network. In addition, we replace the tumor label in the ground truth with the liver label for training the networks for liver CT segmentation. We perform data augmentation on the processed CT scans using the *volumentations*^38^ package to reduce overfitting. The augmentations are randomly applied to the scans and include flips (along the X and Y axis) and transpose operations.

### Implementation details

**Figure 4:**
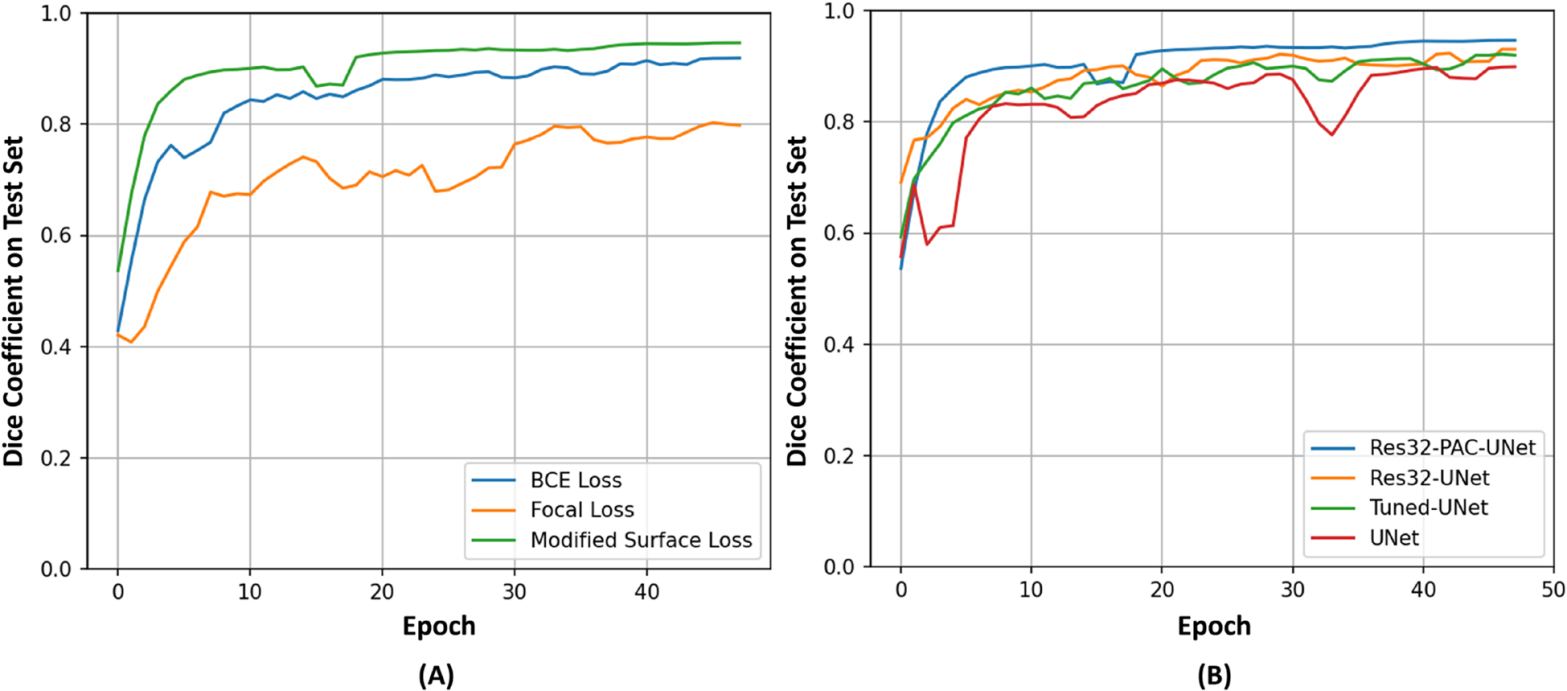
Evolution of DC during the first 50 epochs of training on the test set: (**A**) Res32-PAC-UNet trained with three different loss functions. (**B**) Proposed models trained with modified surface loss function.

To minimize the I/O and computational costs, we pre-process the CT scans and store them in RAM before the training. Additionally, we define the neural networks in Keras and utilize the Tensorflow dataset generator with prefetching to ensure that the neural networks are efficiently fed with augmented scans and the ground truth. The networks are trained for 150 epochs to ensure model convergence (Fig. 4). Adam optimizer (learning rate = 0.0001) and batch size of 1 are used for updating network parameters. We train the Res32-PAC-UNet with three different loss functions to understand their performance impact. The remaining models are trained using the modified surface loss function. The model weights resulting in the highest Dice coefficient on the test set are saved using the Keras callbacks and are utilized during the model evaluation phase.

The models are trained on an HP Z8 workstation with an Intel® Xeon(R) Silver 4216 CPU with a 2.10 GHz base clock (64 cores) and 128 GB of system memory. The workstation also contains an Nvidia Quadro RTX 5000 GPU with 16 GB of VRAM. With the above implementation, the training procedure for the Res32-PAC-UNet model has taken approximately 12 hrs.

### Evaluation metrics

We evaluate the segmentation performance of the networks, mainly by computing area/volume overlap, and class-based accuracy. The metrics can be defined as follows:

*Dice Coefficient (DC) and Symmetric Volume Difference (SVD)*: DC is a region-based metric described as $\frac{2TP}{2TP+FP+FN}$. SVD is the complement of the DC defined as: $1-DC.$

*Intersection over Union (IoU) and Volume Overlap Error (VOE)*: IoU measures the extent of area overlap, which can be described as $\frac{TP}{TP+FN+FP}$. VOE is the complement of the IoU defined as: $1-IoU$

*Specificity*: Specificity measure the ratio of correctly marked negative pixels to the total number of negative pixels in the predicted mask. Specificity is expressed as $\frac{TN}{TN+FP}$.

*Sensitivity*: Sensitivity measures the ratio of correctly marked positive pixels to the total number of positively marked pixels in the predicted segmentation map. Sensitivity is calculated as $\frac{TP}{TP+FN}$.

## Results and discussion

### Impact of loss functions

**Table 1:** Segmentation performance of Res32-PAC-UNet using different loss functions, indicating maximal performance with modified surface loss function.

Loss function	DC	IoU	Sensitivity	Specificity	SVD	VOE
Focal loss	0.898 (0.024)	0.815 (0.038)	0.95 **(0.023)**	**0.998** (0.002)	0.102 (0.024)	0.185 (0.038)
Binary cross entropy	0.949 (0.016)	0.903 (0.028)	0.965 (0.028)	0.997 **(0.001)**	0.051 (0.016)	0.097 (0.028)
Modified surface loss	**0.958** **(0.015)**	**0.92** **(0.026)**	**0.96** (0.026)	0.997 **(0.001)**	**0.042** **(0.015)**	**0.08** **(0.026)**

Significant values are in [bold].

Table 1 shows the segmentation performance summary of the Res32-PAC-UNet model for three different loss functions to evaluate their suitability for liver CT segmentation. The results indicate that the use of binary cross-entropy (BCE) and focal loss leads to sub-optimal segmentation performance, suggesting that the statistical distribution of the classes does not provide sufficient information to the network for achieving high segmentation accuracy. The use of region overlaps and class distribution in the modified surface loss offers an acceptable boost to the segmentation performance, highlighting that area/volume overlap information is essential for segmentation tasks. The modified surface loss further maximizes the segmentation accuracy of the network by employing the boundary loss to refine the edges of the predicted masks. Figure 4 shows the 3-moving average DC of the Res32-PAC-UNet model trained using different loss functions for the first 100 epochs. We apply the moving average to smoothen out the abrupt changes in the DC curve caused by the stochastic update of network weights. It can be deduced that modified surface loss provides high initial segmentation accuracy and provides faster convergence in earlier epochs by attaining an 80% DC in the first five epochs.

The modified surface loss allows the Res32-PAC-UNet to achieve the highest segmentation accuracy among the tested loss functions with accelerated convergence. These results can be explained by the dynamic nature of the loss, which shifts the weights from the combo loss to boundary loss during training. The initial emphasis on the combo loss allows the model to learn the volumetric ROI effectively. In contrast, a definite focus on the boundary loss in the later epochs improves the edge precision of the predicted segmentation masks. In our empirical study, we train the remaining neural networks with the modified surface loss function because of its emphasis on crucial aspects of segmentation masks and superior segmentation accuracy.

**Table 2:** Segmentation performance, disk utilization, and inference time of the proposed models with/without PAC module and related work, trained using modified surface loss.

Model name	DC	IoU	Sensitivity	Specificity	SVD	VOE	Parameter count (Model size in MB)	Inference time (sec)
UNet (2016)	0.919 (0.188)	0.88 (0.182)	0.922 (0.19)	0.997 (0.002)	0.081 (0.188)	0.12 (0.182)	22,575,329 (271)	0.503
Tuned-UNet	0.955 **(0.014)**	0.914 **(0.025)**	0.959 (0.026)	0.997 **(0.001)**	0.045 **(0.014)**	0.086 **(0.025)**	5,644,913 (68)	0.266
Multi-Res-UNet (2020)	0.917 (0.025)	0.848 (0.042)	0.939 (0.036)	0.993 (0.003)	0.083 (0.025)	0.152 (0.042)	4,608,478 (55.8)	0.474
TMD-UNet (2021)	0.923 (0.044)	0.859(0.071)	0.928 (0.071)	0.995 (0.004)	0.077 (0.044)	0.141(0.071)	9,109,969 (110)	4.57
DC-UNet (2021)	0.95 (0.014)	0.905(0.025)	0.959 (0.026)	0.996(0.002)	0.05(0.014)	0.095(0.025)	7,065,285 (85.3)	0.585
Res-UNet++ (2019)	0.956 **(0.014)**	0.916 (0.026)	0.955 (0.028)	0.997 **(0.001)**	0.044 **(0.014)**	0.084 (0.026)	11,786,089 (142)	2.44
Thin16-PAC-UNet	0.946 (0.017)	0.898 (0.03)	0.946 (0.028)	0.997 (0.002)	0.054 (0.017)	0.102 (0.03)	468,737 (5.89)	0.298
Thin32-PAC-UNet	0.95 (0.015)	0.905 (0.026)	0.957 **(0.025)**	0.996 (0.002)	0.05 (0.015)	0.095 (0.026)	1,202,209 (14.81)	0.497
Res16-UNet	0.931 (0.04)	0.873 (0.063)	0.933 (0.037)	0.995 (0.011)	0.069 (0.04)	0.127 (0.063)	**157,345** ** (2.18)**	**0.249**
Res32-UNet	0.954 (0.014)	0.912 (0.025)	0.952 (0.026)	0.997 **(0.001)**	0.046 **(0.014)**	0.088 **(0.025)**	627,521 (7.82)	0.442
Res16-PAC-UNet	0.95 (0.019)	0.905 (0.033)	0.942 (0.029)	**0.998** (0.002)	0.05 (0.019)	0.095 (0.033)	478,849 (6.15)	0.320
Res32-PAC-UNet	**0.958** (0.015)	**0.92** (0.026)	**0.96** (0.026)	0.997 **(0.001)**	**0.042** (0.015)	**0.08** (0.026)	1,227,041(15.1)	0.525

Significant values are in [bold].

### Impact of PAC module on segmentation performance

We conduct a comprehensive empirical study to quantify the impact of using the tuned residual UNet backbone and the PAC module. The performance of the UNet model is established as the baseline for segmentation accuracy and parameter count. Table 2 shows the segmentation performance of the proposed models, including the Res-PAC-UNet, Thin-PAC-UNet, Tuned-UNet, and UNet. The UNet achieves an acceptable DC of 91.9% and sensitivity of 92.2%, surpassing the Multi-Res-UNet^29^ architecture. We modify the Thin-UNet architecture by adding the PAC modules and 3D image compatibility to measure the performance enhancement in the fixed-width backbones. The Thin16-PAC-UNet and Thin32-PAC-UNet outperform the UNet, highlighting the performance gains due to PAC modules in fixed-width lightweight backbones. Next, we add the PAC modules to the tuned Res-UNet backbones. The results show that both Res16-PAC-UNet and Res32-PAC-UNet models significantly boost segmentation performance, with DC increasing from 93.1 to 95% and 95.4 to 95.8%, respectively. Based on this observation, we can deduce that the accuracy gain due to the PAC modules is higher in lighter backbones (i.e., Res16-UNet), thus establishing its importance for lightweight UNet-based backbones. We also note that the Res32-PAC-UNet outperforms the remaining models on all metrics except the specificity.

Figure 4 shows the 3-moving average of DC for the UNet, Tuned-UNet, Res32-UNet, and Res32-PAC-UNet over the first 50 epochs. The UNet experiences slow learning in the first 20 epochs because of its many parameters. On the other hand, the Tuned-UNet model achieves an 80% DC in 10 epochs due to its tuned feature widths throughout the backbone, requiring less training with a limited training set (101 CT scans). The Res32-PAC-UNet architecture shows the fastest learning on the test set by attaining 80% DC in the first five epochs.

The best-in-class segmentation performance and faster convergence of the Res32-PAC-UNet could be associated with the choice of the tuned residual backbone and the usage of PAC modules. The network’s backbone has a fixed width of 32 to reduce the exponential growth of the parameter, minimizing disk utilization. Additionally, it employs residual blocks to improve information and gradient flow, thus allowing the model to learn quickly. The usage of PAC modules over the skip-connections prevents the duplication of low-level features from the encoder to the decoder, replacing them with informative multi-scale volumetric features. Altogether, the Res32-PAC-UNet model overcomes the pitfalls of the conventional UNet and its variants for liver CT segmentation and delivers a better segmentation performance with lower disk utilization.

### Model parameters and storage utilization

**Figure 5:**
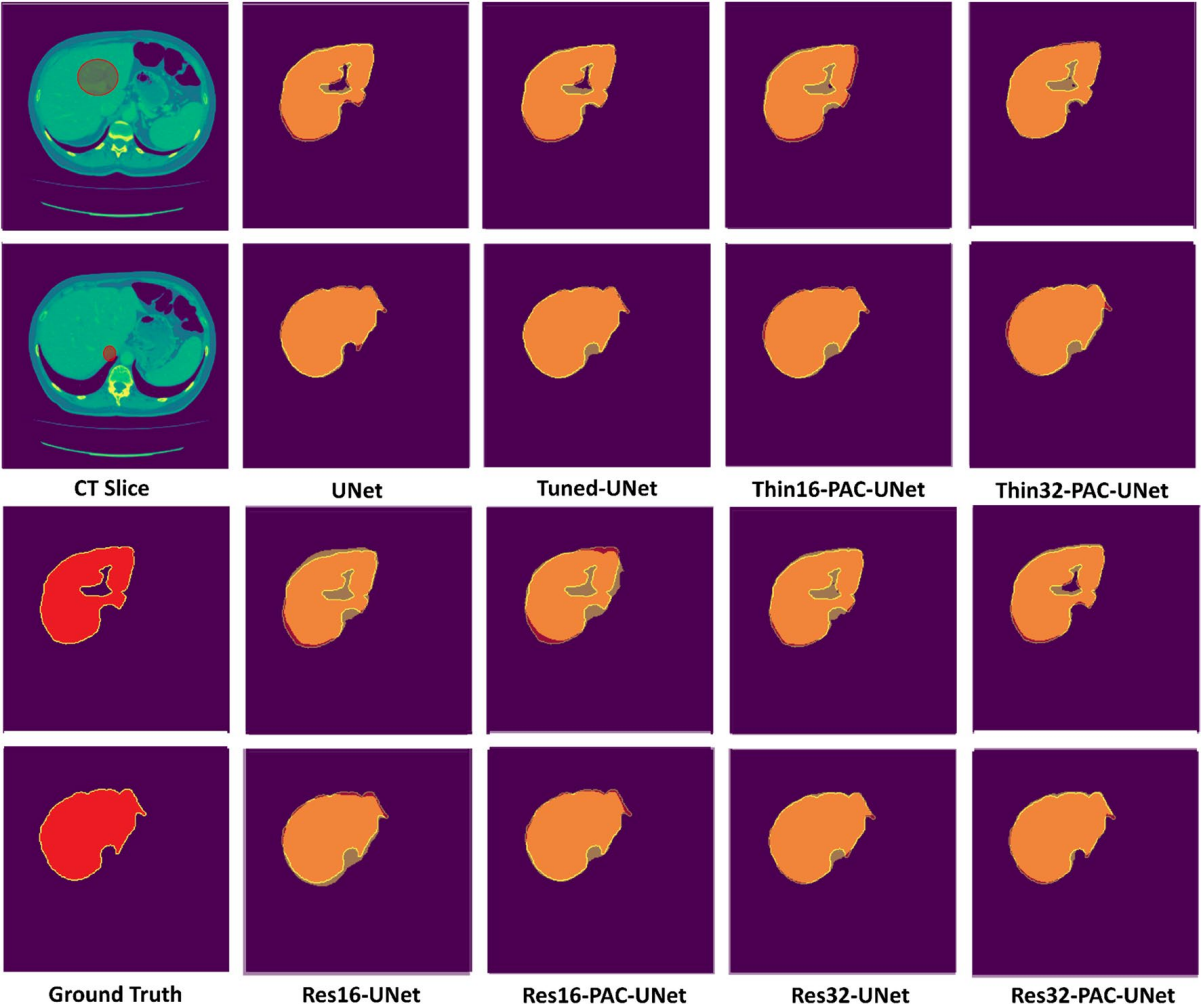
Qualitative comparison of the different segmentation masks generated by the proposed neural networks. The red bounding oval marks the presence of artifacts. The predicted segmentation masks (yellow) are overlaid on the ground truth (red) to highlight region overlap.

Res-PAC-UNet architecture provides the desired tradeoff between segmentation accuracy and disk utilization by varying the feature width in the backbone. Table 2 compares the proposed methods and related work using parameter count and model size. The UNet model provides acceptable segmentation performance but contains nearly 22 million parameters, resulting in a model size of 270 MB. The Tuned-UNet decreases the parameter count and storage space by up to 4$\times$ by limiting the parameters in the early layers of the encoder. The model experiences an improvement in almost all metrics due to its tuned parameter backbone. Specifically, the DC increases from 91.9% to 95.5%, relative to the UNet. This observation suggests that backbones with restricted parameters may outperform UNet in scenarios with limited data. Interestingly, the Thin16-PAC-UNet and Thin32-PAC-UNet approach the segmentation performance of the Tuned-UNet model with nearly 12$\times$, 4.6$\times$ fewer parameters and storage requirements, respectively. The segmentation performance of Thin-PAC-UNets relative to the Tuned-UNet highlights the performance gains due to the PAC module in thin, lightweight architectures.

The Res16-UNet backbone has the lowest parameter count and disk utilization (2.18 MB) while outperforming the baseline UNet model. On the other hand, Res16-PAC-UNet outperforms Thin16-PAC-UNet and matches the performance of Thin32-PAC-UNet in terms of segmentation metrics while having less than half the parameters and models storage requirements. The higher performance of the Res16-PAC-UNet is because of better feature/gradient propagation in the residual backbone relative to the Thin-PAC-UNet architecture. The Res32-PAC-UNet model outperforms all the models in the empirical study while limiting the parameters to 1.2 million and the model size to 15.1 MB. The recently proposed Res-UNet++^28^ architecture has performance closer to the Res32-PAC-UNet, but contains nearly 10$\times$ more parameters. DC-UNet outperforms the Multi-Res-UNet, indicating that the dual-channel pathways in the convolution blocks of the backbone can assist the network in improving the segmentation performance. However, the improvement in the performance of DC-UNet comes at the cost of increased network parameters. Finally, analyzing the results of TMD-UNet suggest that 2D convolution-based neural networks with significant parameter count (i.e., 9.1 million) are unable to capture long-term dependency in the axial direction of CT scan, thereby limiting the segmentation accuracy.

Based on the analysis of segmentation accuracy, parameters count, and model size, we suggest the Res16-PAC-UNet model for machines with disk constraints because it is closer in accuracy to the Tuned-UNet and has a smaller model size (i.e., nearly 11.8$\times$ smaller). Alternatively, when the segmentation accuracy is of utmost importance, the Res32-PAC-UNet achieves the best-in-class accuracy with 18$\times$, 4.6$\times$ fewer parameters than UNet and Tuned-UNet models, respectively.

### Qualitative analysis of segmentation masks

Qualitative analysis is also a crucial aspect of evaluating the proposed neural network’s performance. Figure 5 provides a qualitative comparison of the segmentation masks by overlaying a predicted segmentation mask (in yellow) on the ground truth (in red). In addition, the liver regions excluded from the ground truth have been highlighted in the CT slices. The CT slices highlight the significant obstacles in liver segmentation; for instance, similar image intensities of the neighboring organs and significant boundary variations between adjacent CT slices. UNet architecture accurately predicts the segmentation mask by excluding the areas outside the ROI along the boundary and the center of the liver. The Tuned-UNet architecture slightly over-segments the liver at the edges because of its restricted parameter growth in the backbone. The Thin-PAC-UNet models over-segment the excluded regions at the borders and the center of the liver. Similarly, Res16-UNet and Res16-PAC-UNet models over-segment due to their limited parameters in the backbones. On the other hand, adding the PAC module to the Res32-UNet backbone significantly improves segmentation mask quality, indicating that the fixed-width residual backbone provides more relevant information to the PAC modules relative to Thin-UNet backbones. The Res32-PAC-UNet generates segmentation masks comparable to the UNet while having a fraction of the parameter count.

### Inference time and future directions

Table 2 summarizes the inference time per scan for different networks compared in the empirical study. The UNet architecture takes approximately 0.5 s to generate the predictions. With effective tuning of feature widths in the backbone, the inference time of Tuned-UNet decreases to nearly half (i.e., 0.266 s). Res16-UNet backbone attains the lowest inference of 0.249 s. We can observe that adding PAC modules to the Res-UNet backbones increases the inference times, highlighting its one key limitation. To elaborate, Res32-PAC-UNet has a similar inference time to that of UNet while having 18$\times$ fewer parameters, suggesting that the network-fragmentation and element-wise operations^39^ in PAC modules may impact the run time of a network. Nonetheless, the PAC module allows the lightweight neural networks to maximize their segmentation performance while keeping the disk utilization several times lesser than the UNet and its variants. TMD-UNet and Res-UNet++ take a few seconds to generate a prediction, suggesting that these networks perform heavy computations for predicting liver masks.

In future, we aim to extend this work to determine whether Res-PAC-UNet maintains the same performance for segmentation of liver tumors, vessels, and other organs (i.e., kidney, spleen, and pancreas) across 3D imaging modalities (i.e, CT and MRI). We would also like to construct Res-PAC-UNet like architectures using state-of-the-art computer vision findings that can outperform well-known segmentation models while achieving lower disk utilization and inference times. We plan to achieve this by enhancing the segmentation performance using knowledge distillation by pruning the networks using the TensorRT framework or quantization aware training. Additionally, we think that it may also be beneficial to design networks that achieve acceptable segmentation performance on the CPU.

## Conclusion

In this paper, we propose a novel Res-PAC-UNet architecture that provides a good trade-off between segmentation accuracy and model size. The proposed model employs a tuned fixed-width residual backbone with PAC modules to provide higher segmentation performance with fewer weights and lower disk utilization. The residual backbone restricts the exponential growth rate of the parameters while improving the information and gradient flow, thus assisting the PAC modules present over the skip-connection to extract relevant multi-scale volumetric features. The proposed networks are trained with a modified surface loss function to maximize the segmentation performance. Subsequently, we conduct an empirical study to compare the quantitative and qualitative segmentation performance of the models. We have found that the Res16-PAC-UNet contains fewer weights for liver CT segmentation, while the Res32-PAC-UNet maximizes the segmentation performance. Thus the proposed network provides flexibility to the radiologists to choose models as per their requirements.

## Data Availability

The datasets generated and/or analysed during the current study are available in the medical segmentation decathalon^36^ repository.
