# Publisher Correction: A lightweight neural network with multiscale feature enhancement for liver CT segmentation

**DOI:** 10.1038/s41598-022-20472-5

**Published:** 2022-09-21

**Authors:** Mohammed Yusuf Ansari, Yin Yang, Shidin Balakrishnan, Julien Abinahed, Abdulla Al-Ansari, Mohamed Warfa, Omran Almokdad, Ali Barah, Ahmed Omer, Ajay Vikram Singh, Pramod Kumar Meher, Jolly Bhadra, Osama Halabi, Mohammad Farid Azampour, Nassir Navab, Thomas Wendler, Sarada Prasad Dakua

**Affiliations:** 1 Hamad Medical Corporation, Doha, Qatar; 2 Hamad Bin Khalifa University, Doha, Qatar; 3 Qatar University, Doha, Qatar; 4 Technische Universität München, Munich, Germany; 5 C. V. Raman Global University, Bhubaneswar, India; 6 German Federal Institute for Risk Assessment (BfR), Berlin, Germany; 7 Wake Forest Baptist Medical Center, Winston-Salem, USA

Correction to: *Scientific Reports* 10.1038/s41598-022-16828-6, published online 19 August 2022

The original version of this Article contained errors in References 1 and 2, which were incorrectly given as:

1. Dakua, S. P. journaltitlePerformance divergence with data discrepancy: A review. *Artif. Intell. Rev.*, **40**, 429–455. https://doi.org/10.1007/s10462-011-9289-8 (2013).
2. Dakua, S. P. journaltitleTowards left ventricle segmentation from magnetic resonance images. *IEEE Sens. J.*, **17**, 5971–5981. https://doi.org/10.1109/JSEN.2017.2736641 (2017).

The correct references are listed below:

1. Dakua, S. P. Performance divergence with data discrepancy: A review. *Artif. Intell. Rev.*, **40**, 429–455. https://doi.org/10.1007/s10462-011-9289-8 (2013).
2. Dakua, S. P. Towards left ventricle segmentation from magnetic resonance images. *IEEE Sens. J.*, **17**, 5971–5981. https://doi.org/10.1109/JSEN.2017.2736641 (2017).

The original Article has been corrected.

